# A Letter to the Editors

**Published:** 1818-05

**Authors:** James Curry


THE LONDON

### Medical and Physical Journal.

5 OF VOL. XXXIX.] MAY, 1818. [NO. 231.

"For many fortunate discoveries in medicine, and for the detection of nume-
"rous errors, the world is indebted to the rapid circulation of Monthly
"Journals; and there never existed any work to which the Faculty in
"EUROPE and AMERICA were under deeper obligations than to the
"Medical and Physical Journal of London, now forming a long, but an
"invaluable, series."—RUSH.

*For the London Medical and Physical Journal.*

### *A Letter to the Editors;* by JAMES CURRY, M.D. &c.

I HAVE to apologize for not sooner complying with your very reasonable request,—to produce Dr. Morgan's Inaugural Dissertation on Pus; as you justly remark, that there are few who possess a collection of the earlier Theses published at Edinburgh. I now hasten to repair my omission, and would only remark, as some extenuation of my seeming neglect, that the accompanying volume (one of a very long series, containing *almost all* the Edinburgh Inaugural Dissertations, from their commencement down to the latter part of the last century) was looked out the beginning of last month, and laid upon my writing-table, to be within view, and in readiness to be sent the first opportunity; but, in one of those *periodical sweepings and settings-in-order, which so delight the cleanly housewife, and so annoy the studious sloven,* it was speedily put away, and only recalled to mind by the notice taken of its non-production in your March Number, published the 1st of April.

As I rely upon your candour for a faithful statement of its contents, I shall add nothing upon the question itself, farther than in remarking, that, if, in the first instance, the circumstance of Dr. W. Hunter having denied the Boerhaavian opinion—of pus being produced by a solution of the solid parts, is twisted into an inference, that *he* taught the formation of pus by a *secretory* process; and, in the second, the mere circumstance of John Hunter being, at that time, an assistant to his brother, is to deprive the latter of every claim to improvement or discovery; there is an end to all legitimate adjudication of claims in every similar case; and, Mr. Hunter must, in his turn, surrender his claims to his successors, for no better reason than because they were *his* assistants.

When you have, with the aid of your correspondent, (who has exhibited his *impartiality* by the extracts from Dr. Morgan's Thesis,) substantiated the claim of Mr. Hunter to priority in the doctrine—that pus is a *secretion*, I shall *then*, and not *till then*, feel myself called upon to make good the second part of what I pledged myself for, viz. to demonstrate, that neither Dr. Morgan nor Mr. John Hunter has any claim; in other words, that there is no truth in the doctrine contended for.

*Bridge-street, Blackfriars;*
*April* 2, 1818.

---

*For the London Medical and Physical Journal.*

